# Aspirin-triggered lipoxin A_4 _attenuates LPS-induced pro-inflammatory responses by inhibiting activation of NF-κB and MAPKs in BV-2 microglial cells

**DOI:** 10.1186/1742-2094-8-95

**Published:** 2011-08-10

**Authors:** Yan-Ping Wang, Yan Wu, Long-Yan Li, Jin Zheng, Ren-Gang Liu, Jie-Ping Zhou, Shi-Ying Yuan, You Shang, Shang-Long Yao

**Affiliations:** 1 Department of Anesthesiology and Critical Care, Union Hospital, Tongji Medical College, Huazhong University of Science and Technology, Wuhan, China; 2 Department of Neurology, Union Hospital, Tongji Medical College, Huazhong University of Science and Technology, Wuhan, China; 3 Department of Anatomy, Tongji Medical College, Huazhong University of Science and Technology, Wuhan, China

## Abstract

**Background:**

Microglial activation plays an important role in neurodegenerative diseases through production of nitric oxide (NO) and several pro-inflammatory cytokines. Lipoxins (LXs) and aspirin-triggered LXs (ATLs) are considered to act as 'braking signals' in inflammation. In the present study, we investigated the effect of aspirin-triggered LXA_4 _(ATL) on infiammatory responses induced by lipopolysaccharide (LPS) in murine microglial BV-2 cells.

**Methods:**

BV-2 cells were treated with ATL prior to LPS exposure, and the effects of such treatment production of nitric oxide (NO), inducible nitric oxide synthase (iNOS), interleukin-1β (IL-1β) and tumour necrosis factor-α (TNF-α) were analysed by Griess reaction, ELISA, western blotting and quantitative RT-PCR. Moreover, we investigated the effects of ATL on LPS-induced nuclear factor-κB (NF-κB) activation, phosphorylation of mitogen-activated protein kinases (MAPKs) and activator protein-1 (AP-1) activation.

**Results:**

ATL inhibited LPS-induced production of NO, IL-1β and TNF-α in a concentration-dependent manner. mRNA expressions for iNOS, IL-1β and TNF-α in response to LPS were also decreased by ATL. These effects were inhibited by Boc-2 (a LXA_4 _receptor antagonist). ATL significantly reduced nuclear translocation of NF-κB p65, degradation of the inhibitor IκB-α, and phosphorylation of extracellular signal-regulated kinase (ERK) and p38 MAPK in BV-2 cells activated with LPS. Furthermore, the DNA binding activity of NF-κB and AP-1 was blocked by ATL.

**Conclusions:**

This study indicates that ATL inhibits NO and pro-inflammatory cytokine production at least in part via NF-κB, ERK, p38 MAPK and AP-1 signaling pathways in LPS-activated microglia. Therefore, ATL may have therapeutic potential for various neurodegenerative diseases.

## Background

There is increasing awareness that inflammation may play a role in various neurodegenerative disorders, including Alzheimer's disease, Parkinson's disease, HIV-associated dementia, trauma, multiple sclerosis and stroke [1,2]. Microglial cells are generally considered to be the immune cells of the central nervous system (CNS). They respond to neuronal injury or immunologic challenges with a reaction termed microglial activation. Activated microglial cells can serve diverse beneficial functions essential to neuron survival, which include cellular maintenance and innate immunity [3,4]. However, overactivated microglia can induce significant and highly detrimental neurotoxic effects through excess production of a large array of cytotoxic factors such as superoxide, nitric oxide (NO), tumor necrosis factor-α (TNF-α) and interleukin-1β (IL-1β) [1]. Overactivation of microglia followed by overproduction of neurotoxic factors results in deleterious and progressive neurotoxic consequences [5,6]. In several studies it has been shown that reduction of pro-inflammatory mediators produced by microglia may attenuate the severity of neuronal damage [7]. Therefore, inhibiting inflammatory cytokine production by activated microglia may be useful for preventing neurodegeneration [8-10].

Lipoxins (LXs) are endogenous lipid mediators with potent anti-infiammatory and pro-resolving actions [11]. Of special interest, aspirin can also trigger transcellular biosynthesis of 15-epimers of LX, termed aspirin-triggered LX (ATL) [12], that share the potent anti-infiammatory actions of LX but are more resistant to metabolic inactivation [13]. LXs and ATL elicit multicellular responses via a specific G protein-coupled receptor termed the LXA_4 _receptor (ALX) that has been identified in human [14], mouse [15] and rat [16] tissues. In our previous papers, we evaluated the anti-inflammatory activity of an LXA_4 _analogue, 5(S), 6(R)-LXA_4 _methyl ester, in a rat model of permanent focal cerebral ischemia and focal cerebral ischemia reperfusion [17,18]. Our results showed that this LXA_4 _analogue could attenuate focal ischemia-induced inflammatory responses and inhibit activation of microglia *in vivo*. Expression of functional ALXs was identified in neural stem cells, neurons, astrocytes and microglia [19-23]. Microglial cells are key sensors and versatile effectors in normal and pathologic brain [24]. These findings suggest that microglia may be a target for LXs in brain. However, the effects of LXs on expression of inflammation-related genes and molecular mechanisms in microglia have not been demonstrated.

Lipopolysaccharide (LPS), a component of the outer membrane of Gram-negative bacteria, initiates a number of major cellular responses that play critical roles in the pathogenesis of inflammatory responses and has been commonly used to model proinflammatory and neurotoxic activation of microglia [25,26]. We used LPS as a stimulant of the microglial reactivity in the current study.

In the present study, we investigated the impact of ATL on the infiammatory response induced by LPS in murine microglial BV-2 cells, as well as the signaling pathways involved in these processes. Our data suggest that ATL inhibits NO and pro-inflammatory cytokine production in LPS-activated microglia at least in part via NF-κB, ERK, p38 MAPK and AP-1 signaling pathways.

## Methods

### Cell culture

The immortalized murine microglia cell line BV-2 was purchased from Cell Resource Centre of Peking Union Medical College (Beijing, China) and maintained in Dulbecco's modified Eagle's medium with F12 supplement (DMEM/F12, Gibco, Grand Island, NY) supplemented with 10% fetal bovine serum (Gibco), 100 U/ml penicillin and 100 μg/ml streptomycin at 37°C in a humidified atmosphere of 95% air, 5% CO_2_. Confiuent cultures were passaged by trypsinization. BV-2 cells were seeded onto 96-well plates (10^4 ^cells/well for cell viability assay), 24-well-culture plates (10^5 ^cells/well for ELISA and NO measurement, 10^4 ^cells/well for immunofluorescence), 6-well plates (2.5 × 10^5 ^cells/well for PCR) or 100 mm culture dishes (1.2 × 10^6 ^cells/dish for western blotting and EMSA). Before each experiment, cells were serum-starved for 12 h. BV-2 cells were incubated in the initial experiments with different concentrations (1 nM, 10 nM or 100 nM) of ATL (Cayman Chemical, Ann Arbor, MI), leading to a concentration of 100 nM ATL used in further experiments or vehicle (0.035% ethanol) for 30 min before addition of 100 ng/ml LPS (Escherichia coli O26:B6, Sigma-Aldrich, St. Louis, MO) under serum-free conditions. To investigate the involvement of ALXs in the anti-inflammatory effects of ATL, the cells were treated with 100 μM Boc-2 (Phoenix Pharmaceuticals), a specific receptor antagonist, prior to the treatment with ATL for 30 min.

### RNA isolation, reverse-transcriptase (RT) PCR and real-time PCR

Total RNA was extracted from BV-2 cells with TRIzol reagent (Invitrogen, Carlsbad, CA) according to the manufacturer's protocol. 1.0 μg of total RNA was subjected to oligo-dT-primed RT with ReverTra Ace Kit (Toyobo, Osaka, Japan).

Semi-quantitative PCR was carried out with DNA polymerase (Toyobo) by using specific primers (Invitrogen): 5'-GGCAACTCTGTTGAGGAAAG-3' and 5'-GGCTCTCGGTAGACGAGA-3', which amplify the 423 bp product for ALX1/FPR-rs1; and 5'-GTCAAGATCAACAGAAGAAACC-3' and 5'-GGGCTCTCTCAAGACTATAAGG-3', which amplify 298 bp product for ALX2/FPR2; and 5'-TGGAATCCTGTGGCATCCATGAAAC-3' and 5'-TAAAACGCAGCTCAGTAACAGTCCG-3', which amplify 349 bp product for β-actin. The amplified PCR products were resolved by 2% agarose gel electrophoresis.

Real-time PCR was performed for a quantitative analysis of iNOS, IL-1β and TNF-α mRNA expression using SYBR Green real-time PCR Master Mix (Toyobo) on an MX3000P real-time PCR system (Stratagene). The following primers were used (Invitrogen): 5'-CAGCTGGGCTGTACAAACCTT-3' and 5'- CATTGGAAGTGAAGCGTTTCG-3', which amplify the 95 bp product for iNOS; 5'-CAACCAACAAGTGATATTCTCCATG-3' and 5'- GATCCACACTCTCCAGCTGCA-3', which amplify the 152 bp product for IL-1β; 5'-CATCTTCTCAAAATTCGAGTGACAA-3' and 5'-TGGGAGTAGACAAGGTACAACCC-3', which amplify the 175 bp product for TNF-α; and 5'-TGTCCACCTTCCAGCAGATGT-3' and 5'-AGCTCAGTAACAGTCCGCCTAGA-3', which amplify the 101 bp product for β-actin. Relative gene expression was calculated by the 2^-ΔΔCT ^method [27].

### Cell viability assay

Cell viability was measured by quantitative colorimetric assay with MTT (Sigma-Aldrich), showing the mitochondrial activity of living cells. BV-2 cells in 96-well plates were pretreated with various concentrations of ATL for 30 min and incubated with or without LPS for 24 h in the continued presence of ATL. Upon termination of the experiments, the culture media were aspirated and MTT (0.5 mg/ml) was added to cells and then incubated at 37°C for 4 h. The supernatant was aspirated and dimethyl sulfoxide (Sigma-Aldrich) was added to the wells. Insoluble crystals were dissolved by mixing and the plates were read on an automated Tecan Sunrise absorbance reader, using a test wavelength of 570 nm and a reference wavelength of 630 nm.

### Nitrite measurements

Production of NO was determined by measuring the level of accumulated nitrite, a metabolite of NO in the culture supernatant using Griess reagent (Sigma-Aldrich). After 24 h of treatment with LPS with or without ATL, the culture supernatants were collected and mixed with an equal volume of Griess reagent in 96-well culture plates and incubated at room temperature for 10 min. The absorbance was measured at 540 nm and nitrite concentrations were calculated by reference to a standard curve generated by known concentrations of sodium nitrite.

### ELISA for IL-1β and TNF-α

BV-2 cells in 24-well plates were stimulated for 24 h, and then culture supernatants were harvested. Levels of IL-1β and TNF-α in 100 μl medium were measured by commercial ELISA kits (Boster Biological Technology, Wuhan, China) according to the manufacturer's instructions.

### Immunofluorescence confocal microscopy

For the detection of intracellular location of NF-κB p65, BV-2 cells were cultured on sterile glass cover slips in 24 well plates and treated with ATL and LPS as described above. At various times after the LPS treatment, cells were fixed with 4% paraformaldehyde in PBS and permeabilized with 0.1% Triton X-100 in PBS. After rinsing, cells were blocked with 3% BSA in PBS for 1 h and incubated with rabbit anti-NF-κB p65 antibodies (1:200, Santa Cruz Biotechnology, Santa Cruz) overnight at 4°C. After washing, cells were incubated with FITC-conjugated goat anti-rabbit IgG (1:400, Pierce, Rockford, IL) for 1 h and counterstained with 4, 6-diamidino-2-phenylindole (DAPI, Roche, Shanghai, China) for the identification of nuclei. After washing with PBS, the cover slips were mounted with antifade mounting medium (Beyotime, China) on slides, and the cells were observed with a confocal microscope Olympus Fluoview FV500.

### Protein extraction

For making whole cell lysates, the cells were lysed in radioimmune precipitation assay (RIPA) buffer supplemented with protease inhibitor cocktail (Roche). Nuclear and cytoplasmic fractionations were performed with Proteo JET™ Cytoplasmic and Nuclear Protein Extraction Kit (Fermentas Life Science) according to manufacturer's protocol.

### Western blot analysis

Equal amounts of cytoplasmic, nuclear, or whole cell extracts were electrophoresed on sodium dodecyl sulfate-polyacrylamide gels, and then transferred onto a polyvinylidene difluoride membrane (Millipore). The transformed membrane was blocked for 1 h and incubated with indicated primary antibodies (Santa Cruz Biotechnology) at 4°C overnight. The primary antibodies usedwere as follows: rabbit anti-iNOS (1:500), β-actin (1:1000), p65 (1:1000), Lamin B (1:1000), IκB-α (1:500), ERK1/2 (1:1000), p38 (1:1000), JNK (1:1000) and mouse anti-phosphorylated ERK1/2, p38, JNK antibody (1:1000). The membrane was washed three times with Tris-bufffered saline containing 0.05% Tween 20 (TBST) for 10 min and incubated with anti-rabbit or anti-mouse IgG-horseradish peroxidase (1:5000, Pierce) at room temperature for 1 h. The Supersignal West Pico chemiluminescent substrate system (Pierce) was used to detect immunoreactive bands. The intensity of protein bands after western blotting were quantitated by using Quantity One Version 4.6.3 Image software (Bio-Rad) and normalized against proper loading controls.

### Electrophoretic mobility shift assay (EMSA)

Nuclear extracts were prepared as described above. Oligonucleotides corresponding to the NF-κB (5'-AGTTGAGGGGACTTTCCCAGGC-3') and AP-1 (5'-CGCTTGATGAGTCAGCCGGAA-3') binding site consensus sequences were synthesized and end-labeled with biotin by Invitrogen. EMSAs were performed using the LightShift chemiluminescent EMSA kit (Pierce). Briefly, 20 fmol of biotin-labeled, double strand probe was incubated for 20 min at room temperature in 20 μl of EMSA binding buffer containing 2.5% glycerol, 5 mM MgCl_2_, 50 ng/μl poly (dI-dC), 0.05% Nonidet P-40, and 6 μg of nuclear proteins. For competition EMSA, 200-fold (4 pmol) excess unlabeled, double strand probe was added to the binding reaction. The DNA-nuclear protein complexes were resolved by electrophoresis in 6% nondenaturing polyacrylamide gel in 0.5 × Tris-borate-EDTA (TBE) buffer at 100 V. Gels were then electroblotted onto Hybond nylon membranes (GE Healthcare) at 380 mA for 50 min. The membranes were then cross-linked for 15 min with the membrane face down on a transilluminator at 312 nm, and the biotinylated protein-DNA bands were detected with HRP-conjugated streptavidin using the chemiluminescent nucleic acid detection system (Pierce).

### Statistical analysis

Data are expressed as means ± SEM of the indicated number of independent experiments. Changes in IκB protein levels were analyzed by two-way ANOVA (treatment and time). All other data were analyzed by one-way ANOVA. Least significant difference (LSD) post hoc test was used for multiple comparisons. Statistical analysis was performed using the SPSS software version 17.0 (SPSS Inc., Chicago, IL, USA). *P *< 0.05 was considered statistically significant.

## Results

### ALXs are expressed in BV-2 microglial cells

**Figure 1:**
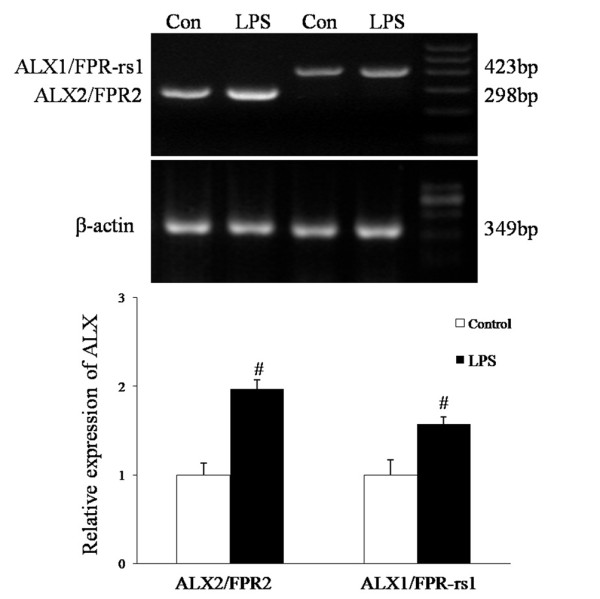
**ALX expression in murine BV-2 microglial cells**. BV-2 cells were incubated with or without LPS (100 ng/ml) at 37°C for 6 h. Total RNA was extracted and the expressions of ALX1/FPR-rs1 and ALX2/FPR2 mRNAs were examined by RT-PCR. β-Actin was used as a loading control. RT-PCR products were electrophoresed on 2% agarose gel. Quantification of ALX1/FPR-rs1 and ALX2/FPR2 mRNAs levels was performed by densitometric analysis. Each value represents the mean ± SEM for three independent experiments. *^#^P <*0.05 compared with control.

Using RT-PCR, we showed that both ALX1/FPR-rs1 and ALX2/FPR2 were expressed in BV-2 microglial cells. The mRNA expression levels of these two receptors were significantly enhanced when the cells were exposed to LPS (100 ng/ml) for 6 h (Figure 1).

### ATL inhibits LPS-induced NO, IL-1β and TNF-α production in BV-2 cells

**Figure 2:**
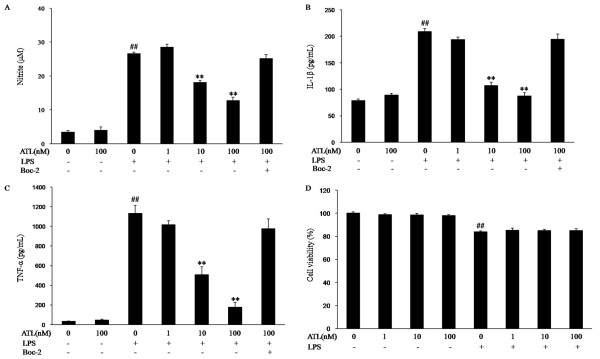
**Inhibition of NO, IL-1β and TNF-α production by ATL in LPS-stimulated BV-2 cells**. BV-2 cells were pretreated with vehicle (0.035% ethanol) or various concentrations of ATL (1, 10 and 100 nM) for 30 min in the absence or presence of 100 μM Boc-2 (30 min before ATL treatment), a lipoxin receptor antagonist, followed by stimulated with LPS (100 ng/ml) for 24 h. (A) Nitrite content was measured using the Griess reaction. The concentration of IL-1β (B) and TNF-α (C) in culture media was measured using a commercial ELISA kit. (D) Cell viability was assessed by MTT assay, and the results are expressed as the percentage of surviving cells compared to control cells. Each value represents the mean ± SEM for three independent experiments. **P <0.01 compared with LPS in the absence of ATL; *^##^P <*0.01 compared with vehicle.

Initially, we evaluated the effects of ATL on NO, IL-1β and TNF-α production in LPS-stimulated BV-2 microglia. BV-2 cells were incubated with vehicle or different concentrations of ATL (1, 10 and 100 nM) for 30 min and stimulated with 100 ng/ml LPS for 24 h. To determine NO production, we measured nitrite released into the culture medium using the Griess reagent. Stimulation of BV-2 cells with LPS markedly increased (about 7.5-fold) NO production, compared with that generated under control conditions. Pretreatment with ATL significantly inhibited this increase in a concentration-dependent manner (Figure 2A).

We then tested whether ATL reduces the production of LPS-induced pro-inflammatory cytokines IL-1β and TNF-α using ELISA. As shown in Figure 2B and 2C, stimulation of BV-2 cells with LPS led to a significant increase in the levels of IL-1β and TNF-α in the cell-conditioned media after 24 h. Pretreatment of BV-2 cells with ATL significantly inhibited the LPS-induced IL-1β and TNF-α production, concentration dependently.

To evaluate the role of the ALXs in the anti-inflammatory effects of ATL, BV-2 cells were treated with an ALX antagonist, Boc-2 (100 μM, 30 min) prior to treatment with ATL. Pretreatment with Boc-2 inhibited these effects in response to ATL (Figure 2).

To exclude the possibility that the decrease in the NO and cytokines levels was simply due to the cytotoxicity of the drug, cell viability was evaluated. The cytotoxic effects of ATL in BV-2 cells were evaluated in the absence or presence of LPS using MTT assays. ATL (1, 10 and 100 nM) and vehicle did not affect cell viability (Figure 2D). When cells were treated with 100 ng/ml LPS only, a decrease in viability was detected compared with the control cells. However, cells pretreated with ATL for 30 min showed no significant increase compared with cells that were treated with LPS only (Figure 2D). Therefore, the inhibitory effect of ATL on LPS-induced, inflammation-related responses in activated BV-2 cells was not the result of ATL effects on cell survival.

### ATL inhibits mRNA expressions of iNOS, IL-1β, and TNF-α

**Figure 3:**
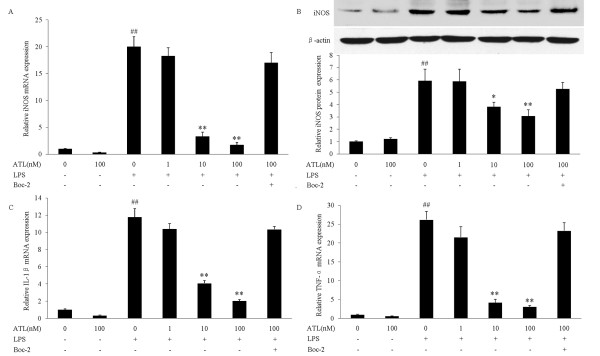
**Inhibition of iNOS, IL-1β and TNF-α mRNA expression by ATL in LPS-stimulated BV-2 cells**. BV-2 cells were pretreated with ATL (1, 10 and 100 nM) for 30 min in the absence or presence of 100 μM Boc-2 (30 min before ATL treatment) followed by incubation with LPS (100 ng/ml). Total RNA was prepared 6 h later and expression of iNOS (A), IL-1β (C) and TNF-α (D) mRNA was measured by real-time PCR. Levels of each mRNA were normalized to those of the house-keeping gene β-actin. The expression of iNOS protein was assessed by western blot analysis 24 h later (B). Detection of β-actin was also carried out to confirm the equal loading of proteins. Each value represents the mean ± SEM for three independent experiments.*P < 0.05 compared with LPS in the absence of ATL;**P <0.01 compared with LPS in the absence of ATL; *^##^P *< 0.01 compared with vehicle.

To find out whether ATL suppresses iNOS, IL-1β and TNF-α expression at the transcriptional level, BV-2 cells were incubated for 30 min with the indicated concentrations of ATL and then incubated with 100 ng/ml LPS for 6 h. The relative amounts of iNOS, IL-1β and TNF-α mRNA were determined by real-time RT-PCR. As anticipated, LPS induced a marked increase in iNOS, IL-1β and TNF-α mRNA in BV-2 cells, about 20, 11, 26-fold increase, respectively (Figure 3). Pretreatment with ATL reduced LPS-induced up-regulation of iNOS, IL-1β and TNF-α mRNA levels in a dose-dependent manner (Figure 3). The inhibitory effects of ATL on LPS-induced iNOS mRNA up-regulation were accompanied by attenuation of iNOS protein induction (Figure 3B). ATL inhibition of LPS-induced expression of iNOS, IL-1β and TNF-α was reversed after pre-exposure of BV-2 cells to the ALX antagonist Boc-2 (100 μM) for 30 min (Figure 3). Taken together, our current data prove that ATL inhibits the inflammatory activation of BV-2 microglia cells with respect to NO production and pro-inflammatory cytokine expression.

### ATL inhibits nuclear translocation of NF-κB and degradation of IκB-α

**Figure 4:**
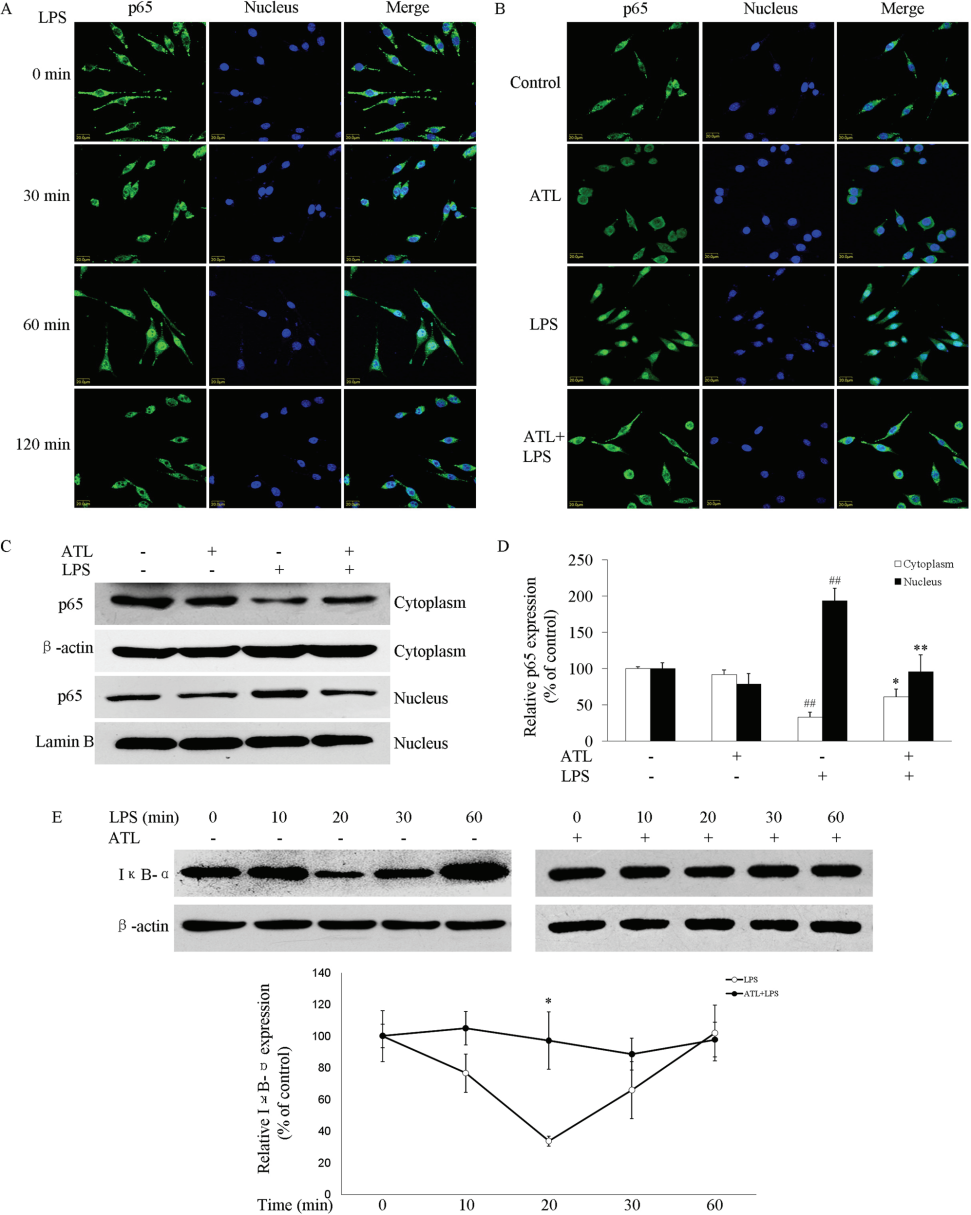
**Inhibition of the nuclear accumulation of the NF-κB p65 subunit and degradation of IκB-α by ATL in LPS-stimulated BV-2 microglial cells**. (A) BV-2 cells were stimulated with 100 ng/ml LPS for the indicated times. Subcellular localization of p65 subunit was evaluated using an anti-p65 antibody and a FITC-labelled anti-rabbit IgG antibody. DNA was stained using DAPI to visualize nuclei, and cells were visualized using laser confocal scanning microscopy. Note that nuclear translocation of the p65 subunit is not complete, but that part of the cytoplasmic p65 is translocated to the nucleus so that the distinction between the nucleus and the cytoplasm blurs. This is obvious 60 min after activation. (B) BV-2 cells were stimulated with 100 ng/ml LPS in the absence or presence of 100 nM ATL that had been added 30 min before activation. Subcellular location of the p65 subunit was tested using immunofluorescence assay 60 min after activation. (C) BV-2 cells were stimulated as in B. Cytoplasmic and nuclear extracts were separated by SDS-PAGE and immunoblotted with anti-p65 antibody. The same extracts were re-electrophoresed and immunoblotted for β-actin or lamin B to monitor loading. A representative result from three independent experiments is shown. (D) Quantification of cytoplasmic and nuclear p65 bands from the experiments in C was normalized by β-actin or lamin B. (E) BV-2 cells were pretreated with vehicle or 100 nM ATL for 30 min and stimulated with LPS (100 ng/ml). Levels of IκB-α in cellular lysates were analyzed using western blotting at indicated times. Quantification of IκB-α protein levels was performed by densitometric analysis. Data are presented as mean ± SEM for three independent experiments.*P < 0.05 compared with LPS in the absence of ATL;**P <0.01 compared with LPS in the absence of ATL; *^##^P *< 0.01 compared with vehicle.

Because ATL reduced the transcriptional activation of iNOS, IL-1β and TNF-α genes, it is likely that it blocks signaling events involved in transcriptional activation of these genes. Expression of iNOS and cytokines genes requires NF-κB activation and nuclear translocation to interact with DNA. Therefore, the involvement of NF-κB nuclear translocation in ATL-induced suppression of NO and cytokines was examined by fluorescence microscopy. LPS stimulation caused obvious translocation of NF-κB p65 from the cytoplasm into the nucleus 60 min after activation (Figure 4A), whereas the presence of 100 nM ATL reduced this (Figure 4B). To further verify the p65 nuclear translocation data, we analyzed the cells by western blotting and found that pretreatment of cells with 100 nM ATL prevented p65 nuclear localization induced by LPS (Figure 4C and 4D).

To address the possibility that the impaired nuclear translocation of p65 was due to inhibition of degradation of IκB-α, we examined the effect of ATL on IκB-α degradation induced by LPS. Western blot analysis showed that LPS-induced degradation of IκB-α was significantly reversed by 100 nM ATL in BV-2 cells (Figure 4E).

### ATL inhibits LPS-induced ERK and p38 MAPK activation

**Figure 5:**
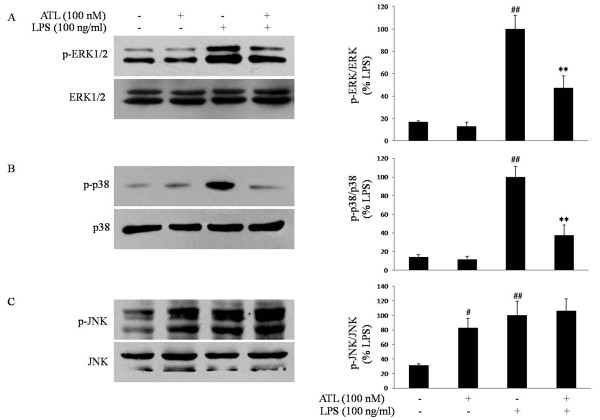
**Inhibition of LPS-induced phosphorylation of ERK and p38 MAPK in BV-2 microglial cells**. BV-2 cells were stimulated with 100 ng/ml LPS in the absence or presence of 100 nM ATL that had been added 30 min before activation. Levels of ERK and phosphorylated ERK (A), p38 and phosphorylated p38 (B), and JNK and phosphorylated JNK (C) were analyzed using western blotting 30 min after stimulation with LPS. The figures show representative results of three independent experiments. Each bar represents the means ± SEM. **P < 0.01 compared with LPS in the absence of ATL;*^#^P <*0.05 compared with vehicle; *^##^P *< 0.01 compared with vehicle.

Along with NF-κB, MAPKs are known to play an important role in the signaling pathways that induce proinfiammatory cytokines and iNOS in glial cells [28]. To investigate whether the inhibition of infiammation by ATL is regulated by the MAPK pathway, we examined the effects of ATL on LPS-induced phosphorylation of ERK, p38 MAPK and JNK in BV-2 microglia by western blot analysis. Cells were pretreated with 100 nM ATL for 30 min and then incubated with 100 ng/ml LPS for 30 min. The 30-min treatment of LPS was determined to be optimal in a preliminary study that examined MAPK phosphorylation at 0, 10, 20, 30, and 60 min after LPS treatment (data not shown). ATL (100 nM) markedly inhibited ERK and p38 MAPK activation, while phosphorylation of JNK was not affected (Figure 5A-C). Strikingly, ATL could induce JNK phosphorylation without effect on ERK and p38 MAPK activity.

### ATL inhibits LPS-induced NF-κB and AP-1 DNA binding activity

**Figure 6:**
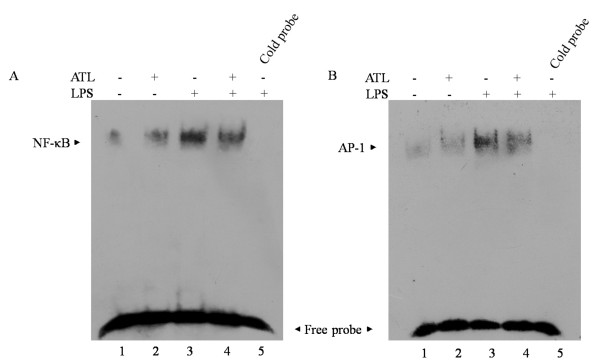
**Inhibitory effects of ATL on NF-κB and AP-1 DNA-binding activities**. BV-2 cells were pretreated with ATL for 30 min and stimulated with LPS for 1 h. Nuclear extracts were prepared and used to analyze NF-κB (A) and AP-1 (B) DNA-binding activity by EMSA, as described in Methods. Binding specificity was confirmed by unlabelled probe (100-fold in excess; lane 5) to compete with labelled oligonucleotide. The arrow indicates the NF-κB or AP-1 binding complex. Free-labelled probes are also indicated by an arrow. Results were confirmed by three independent experiments.

To determine the effects of ATL on transcription factor signaling pathways that might mediate LPS-induced proinfiammatory cytokines production, EMSA was performed. BV-2 cells were pretreated with vehicle and 100 nM ATL for 30 min before stimulation with LPS (100 ng/ml) for 1 h. NF-κB and AP-1 binding activities were induced by LPS treatment (Figure 6A and 6B, lane 3). Binding specificity was verified by incubating nuclear extracts from LPS-stimulated BV-2 cells with excess unlabeled specific competitor oligonucleotide probe (Figure 6A and 6B, lane 5). Pretreatment with ATL markedly reduced the LPS-induced DNA-binding activity of NF-κB and AP-1 (Figure 6A and 6B, lane 4).

## Discussion

Our present data provide the first evidence that ATL inhibits the infiammatory activation of microglia. To date, two separate LXA_4 _receptors (ALX1/FPR-rs1 and ALX2/FPR2) have been identified in mice [15,29]. Mouse ALX2/FPR2 is expressed by neutrophils, monocytes, macrophages, dendritic cells, and microglial cells, and its transcripts are detected at high levels in spleen and lung [30]. ALX1/FPR-rs1 and ALX2/FPR2 are both expressed in the mouse pituitary gland, hypothalamic tissue and vomeronasal organ [31,32]. As demonstrated by RT-PCR analysis, ALX1/FPR-rs1 and ALX2/FPR2 are both expressed in BV-2 microglial cells. ATL reduced LPS-induced production of NO, IL-1β and TNF-α in BV-2 microglial cells. This is a receptor-mediated effect as it disappeared when microglial cells were pretreated with Boc-2 before ATL treatment. Quantitative PCR analysis showed that ATL markedly suppresses iNOS, IL-1β and TNF-α gene expression in BV-2 microglia cells. Similarly, this effect was abrogated by the use of Boc-2. NF-κB, ERK and p38 MAPK pathways are at least partly involved in the anti-infiammatory mechanisms of ATL in BV-2 cells. Thus, ATL is a promising agent for preventing and treating neuroinflammation and may be useful for mitigating a dysregulated linkage between the immune system and brain.

Although microglial activation has important repairative functions in the CNS, microglial cell activation in infection, infiammation, or injury may go beyond control and eventually produce detrimental effects that override the beneficial effects. Activation of microglia leads to release of various toxic molecules such as superoxide, NO, IL-1β and TNF-α, contributing to neuronal damage in various neurodegenerative disorders [1].

LX possesses dual anti-inflammatory and pro-resolution activities that have been demonstrated in a multitude of acute and chronic inflammatory conditions [11]. Previously, LXA_4_, ATL and their stable analogues have been shown to play a major role in important functional properties of the central nervous system, such as neural stem cell proliferation and differentiation, pain, and cerebral ischemia [17-19,33]. In primary murine microglia or N9 microglial cells, expression of ALX2/FPR2 has been identified and is up-regulated by inflammatory stimuli [20,21]. In the present study, the expression of ALX2/FPR2 and another murine high-affinity ALX1/FPR-rs1 were confirmed in BV-2 microglial cells. These findings suggest that ATL could work as a modulator of the inflammatory reaction of the brain immune system, eventually acting as a microglial activation repressor.

NO and pro-infiammatory cytokines such as IL-1β and TNF-α are known to be important mediators in the process of infiammation. These proinfiammatory mediators are thought to be responsible for some of the harmful effects of brain injuries and diseases, including ischemia, Alzheimer's disease, Parkinson's disease and multiple sclerosis [34]. Under various pathological conditions associated with infiammation, large amounts of NO are produced in the brain as a result of the induced expression of iNOS in glial cells [35]. High levels of NO exert their toxic effects through multiple mechanisms, including lipid peroxidation, mitochondrial damage, protein nitration and oxidation, depletion of antioxidant reserves, activation or inhibition of various signaling pathways, and DNA damage [35]. Therefore, the effect of ATL on NO production and iNOS expression in LPS-stimulated microglia cells was examined. As shown in previous research [36,37], NO is produced at low levels in unstimulated microglia. Stimulation of BV-2 microglial cells with LPS induced strong NO production and iNOS expression. The magnitude of the NO/iNOS response to LPS in BV-2 microglial cells is different in different studies with different concentrations as well as durations of LPS treatment. In the present study, ATL markedly reduced NO production and mRNA and protein expression of iNOS in dose-dependent manners without significant cytotoxicity. This indicates that inhibition of NO production by ATL is a result of inhibition of iNOS gene expression. Previous studies also have shown that LXA_4 _and ATL analogues inhibit LPS-induced NO production and peroxynitrite formation in human leukocytes [38] and in mouse lung [39].

Pro-infiammatory cytokines produced by activated microglia, including IL-1β and TNF-α, play an important role in the process of neuroinfiammatory diseases [34]. IL-1β is a potent pro-infiammatory cytokine that acts through IL-1 receptors found on numerous cell types, including neurons and microglia. TNF-α can cause cell death directly by binding to neuronal TNF receptors linked to death domains that activate caspase-dependent apoptosis [40] or by potentiating glutamate release, thereby enhancing excitotoxicity [41]. IL-1β and TNF-α also drive self-propagating cycles of microglial activation and neuroinflammation by inducing activation of NF-κB, cytokine generation and further activation of NF-κB. Thus, inhibition of cytokine production or function serves as a key mechanism in the control of neurodegeneration. Our results showed that ATL markedly attenuates the production of IL-1β and TNF-α, and their mRNA expressions; induced by LPS in BV-2 cells. Consistent with our findings, similar results have shown that LXA_4 _and ATL inhibit LPS-induced production of IL-1β and TNF-α in uvea and in macrophages and endothelial cells [42-44].

In subsequent studies, we found that ATL has a strong inhibitory effect on infiammatory signaling pathways that include NF-κB and MAPK/AP-1. NF-κB activity increases in acute neurodegenerative disorders such as stroke, severe epileptic seizures, and traumatic brain injury; and in chronic neurodegenerative conditions, including Alzheimer's disease, Parkinson's disease, Huntington disease, and amyotrophic lateral sclerosis [45]. In general, activation of NF-κB in microglia contributes to neuronal injury and promotes the development of neurodegenerative disorders [45]. NF-κB is known as a pleiotropic regulator of various genes involved in the production of many proinfiammatory cytokines and enzymes. NF-κB is also a central regulator of microglial responses to activating stimuli, including LPS and cytokines [46]. In this study, ATL was able to inhibit the LPS-evoked degradation of IκB-α, nuclear translocation of NF-κB p65 and the DNA-binding activities of NF-κB in BV-2 cells. Previous studies have shown that LXs reduce nuclear translocation of NF-κB in human neutrophils, mononuclear leukocytes [38] and macrophages [43]. It has also been reported that ATLs reduce NF-κB-mediated transcriptional activation in an ALX-dependent manner, and inhibit the degradation of IκB [47]. Therefore, induction of anti-inflammatory responses by LXs may be dependent on the NF-κB signaling pathway.

In addition, LPS also activates MAPK pathways which lead to the induction of another transcription factor, AP-1. MAPKs are a group of signaling molecules that appear to play key roles in infiammatory processes [48]. We found that phosphorylation of ERK and p38 MAPK in response to LPS is decreased by ATL treatment. Our results also show that ATL treatment of BV-2 microglia results in decreased DNA-binding activities of AP-1 following LPS stimulation. This observation is in line with studies in mesangial cells, endothelial cells, neutrophils, fibroblasts and T cells, which have shown that ERK and/or p38 MAPK activation is attenuated in the presence of LXs [42,49-51]. In the present study, ATL failed to inhibit LPS-induced phosphorylation of JNK. A previous study in primary astrocytes found that an ATL analogue prevents ATP-evoked JNK phosphorylation, but has no effect on TNF-α-induced JNK phosphorylation [33]. Strikingly, our results show that ATL induces JNK phosphorylation, but has no effect on ERK and p38 MAPK activity. In another study, LXA_4 _attenuated microvascular fluid leaks caused by LPS partly mediated by the JNK signaling pathway [52]. LXA_4 _and ATL analogues could promote ERK phosphorylation in macrophages and monocytes [53,54]. The reasons for these discrepancies are mainly due to differences in experimental models, cell types and stimulators.

## Conclusions

In summary, our results show that ATL inhibits release of NO and pro-inflammatory cytokines in a concentration-dependent manner. Moreover, ATL acts at the level of transcription in LPS-stimulated microglia. A possible mechanism for this effect involves ATL's ability to activate a signaling cascade that results in repression of NF-κB, ERK and p38 MAPK activation in activated microglia. Given the fact that microglial activation contributes to the pathogenesis of neurodegenerative diseases, ATL may be considered as a potential therapeutic agent for neurodegenerative diseases involving neuroinflammation.

## Abbreviations

ALX: lipoxin A_4 _receptor; AP-1: activator protein-1; ATL: aspirin-triggered lipoxin A_4_; CNS: central nervous system; EMSA: Electrophoretic mobility shift assay; ERK: extracellular signal-regulated kinase; IL: interleukin; iNOS: inducible nitric oxide synthase; IκB: inhibitor of κB; JNK: c-jun N-terminal kinase; LPS: lipopolysaccharide; LX: lipoxin; LXA_4_: lipoxin A_4_; MAPK: mitogen-activated protein kinase; NF-κB: nuclear factor-κB; RIPA: radioimmune precipitation assay buffer

## Competing interests

The authors declare that they have no competing interests.

## Authors' contributions

YPW, YW and LYL performed the experiments and analyzed the data. JZ, RGL, and JPZ provided useful advice and reviewed the manuscript. YS conceived the study, participated in its design and coordination, and wrote the manuscript. SYY and SLY oversaw the experimental design and edited the manuscript. All authors of this paper have read and approved the final version the manuscript.

## References

[B1] Block ML Zecca L Hong JS Microglia-mediated neurotoxicity: uncovering the molecular mechanisms Nat Rev Neurosci 2007 8 57 69 10.1038/nrn2038 17180163

[B2] Amor S Puentes F Baker D van der Valk P Inflammation in neurodegenerative diseases Immunology 2010 129 154 169 10.1111/j.1365-2567.2009.03225.x 20561356 PMC2814458

[B3] Brandenburg LO Jansen S Wruck CJ Lucius R Pufe T Antimicrobial peptide rCRAMP induced glial cell activation through P2Y receptor signalling pathways Mol Immunol 2010 47 1905 1913 10.1016/j.molimm.2010.03.012 20392497

[B4] Brandenburg LO Varoga D Nicolaeva N Leib SL Wilms H Podschun R Wruck CJ Schroder JM Pufe T Lucius R Role of glial cells in the functional expression of LL-37/rat cathelin-related antimicrobial peptide in meningitis J Neuropathol Exp Neurol 2008 67 1041 1054 10.1097/NEN.0b013e31818b4801 18957897

[B5] Polazzi E Contestabile A Reciprocal interactions between microglia and neurons: from survival to neuropathology Rev Neurosci 2002 13 221 242 10.1515/REVNEURO.2002.13.3.221 12405226

[B6] Streit WJ Conde JR Fendrick SE Flanary BE Mariani CL Role of microglia in the central nervous system's immune response Neurol Res 2005 27 685 691 16197805 10.1179/016164105X49463a

[B7] Ramirez BG Blazquez C Gomez del Pulgar T Guzman M de Ceballos ML Prevention of Alzheimer's disease pathology by cannabinoids: neuroprotection mediated by blockade of microglial activation J Neurosci 2005 25 1904 1913 10.1523/JNEUROSCI.4540-04.2005 15728830 PMC6726060

[B8] Jin R Yang G Li G Inflammatory mechanisms in ischemic stroke: role of inflammatory cells J Leukoc Biol 2010 87 779 789 10.1189/jlb.1109766 20130219 PMC2858674

[B9] Block ML Hong JS Microglia and inflammation-mediated neurodegeneration: multiple triggers with a common mechanism Prog Neurobiol 2005 76 77 98 10.1016/j.pneurobio.2005.06.004 16081203

[B10] Lull ME Block ML Microglial activation and chronic neurodegeneration Neurotherapeutics 2010 7 354 365 10.1016/j.nurt.2010.05.014 20880500 PMC2951017

[B11] Serhan CN Chiang N Van Dyke TE Resolving inflammation: dual anti-inflammatory and pro-resolution lipid mediators Nat Rev Immunol 2008 8 349 361 10.1038/nri2294 18437155 PMC2744593

[B12] Claria J Serhan CN Aspirin triggers previously undescribed bioactive eicosanoids by human endothelial cell-leukocyte interactions Proc Natl Acad Sci USA 1995 92 9475 9479 10.1073/pnas.92.21.9475 7568157 PMC40824

[B13] Serhan CN Savill J Resolution of inflammation: the beginning programs the end Nat Immunol 2005 6 1191 1197 10.1038/ni1276 16369558

[B14] Fiore S Maddox JF Perez HD Serhan CN Identification of a human cDNA encoding a functional high affinity lipoxin A4 receptor J Exp Med 1994 180 253 260 10.1084/jem.180.1.253 8006586 PMC2191537

[B15] Takano T Fiore S Maddox JF Brady HR Petasis NA Serhan CN Aspirin-triggered 15-epi-lipoxin A4 (LXA4) and LXA4 stable analogues are potent inhibitors of acute inflammation: evidence for anti-inflammatory receptors J Exp Med 1997 185 1693 1704 10.1084/jem.185.9.1693 9151906 PMC2196289

[B16] Chiang N Takano T Arita M Watanabe S Serhan CN A novel rat lipoxin A4 receptor that is conserved in structure and function Br J Pharmacol 2003 139 89 98 10.1038/sj.bjp.0705220 12746227 PMC1573822

[B17] Wu Y Ye XH Guo PP Xu SP Wang J Yuan SY Yao SL Shang Y Neuroprotective effect of lipoxin A(4) methyl ester in a rat model of permanent focal cerebral ischemia J Mol Neurosci 2010 42 226 234 10.1007/s12031-010-9355-8 20401639

[B18] Ye XH Wu Y Guo PP Wang J Yuan SY Shang Y Yao SL Lipoxin A4 analogue protects brain and reduces inflammation in a rat model of focal cerebral ischemia reperfusion Brain Res 2010 1323 174 183 20138164 10.1016/j.brainres.2010.01.079

[B19] Wada K Arita M Nakajima A Katayama K Kudo C Kamisaki Y Serhan CN Leukotriene B4 and lipoxin A4 are regulatory signals for neural stem cell proliferation and differentiation FASEB J 2006 20 1785 1792 10.1096/fj.06-5809com 16940150

[B20] Cui YH Le Y Zhang X Gong W Abe K Sun R Van Damme J Proost P Wang JM Up-regulation of FPR2, a chemotactic receptor for amyloid beta 1-42 (A beta 42), in murine microglial cells by TNF alpha Neurobiol Dis 2002 10 366 377 10.1006/nbdi.2002.0517 12270697

[B21] Chen K Iribarren P Hu J Chen J Gong W Cho EH Lockett S Dunlop NM Wang JM Activation of Toll-like receptor 2 on microglia promotes cell uptake of Alzheimer disease-associated amyloid beta peptide J Biol Chem 2006 281 3651 3659 16339765 10.1074/jbc.M508125200

[B22] Braun BJ Slowik A Leib SL Lucius R Varoga D Wruck CJ Jansen S Podschun R Pufe T Brandenburg LO The formyl peptide receptor like-1 and scavenger receptor MARCO are involved in glial cell activation in bacterial meningitis J Neuroinflammation 2011 8 11 10.1186/1742-2094-8-11 21299846 PMC3040686

[B23] Decker Y McBean G Godson C Lipoxin A4 inhibits IL-1beta-induced IL-8 and ICAM-1 expression in 1321N1 human astrocytoma cells Am J Physiol Cell Physiol 2009 296 C1420 1427 10.1152/ajpcell.00380.2008 19357230

[B24] Hanisch UK Kettenmann H Microglia: active sensor and versatile effector cells in the normal and pathologic brain Nat Neurosci 2007 10 1387 1394 10.1038/nn1997 17965659

[B25] Qin L Wu X Block ML Liu Y Breese GR Hong JS Knapp DJ Crews FT Systemic LPS causes chronic neuroinflammation and progressive neurodegeneration Glia 2007 55 453 462 10.1002/glia.20467 17203472 PMC2871685

[B26] Wilms H Sievers J Rickert U Rostami-Yazdi M Mrowietz U Lucius R Dimethylfumarate inhibits microglial and astrocytic inflammation by suppressing the synthesis of nitric oxide, IL-1beta, TNF-alpha and IL-6 in an in-vitro model of brain inflammation J Neuroinflammation 2010 7 30 10.1186/1742-2094-7-30 20482831 PMC2880998

[B27] Livak KJ Schmittgen TD Analysis of relative gene expression data using real-time quantitative PCR and the 2(-Delta Delta C(T)) Method Methods 2001 25 402 408 10.1006/meth.2001.1262 11846609

[B28] Koistinaho M Koistinaho J Role of p38 and p44/42 mitogen-activated protein kinases in microglia Glia 2002 40 175 183 10.1002/glia.10151 12379905

[B29] Vaughn MW Proske RJ Haviland DL Identification, cloning, and functional characterization of a murine lipoxin A4 receptor homologue gene J Immunol 2002 169 3363 3369 12218158 10.4049/jimmunol.169.6.3363

[B30] Ye RD Boulay F Wang JM Dahlgren C Gerard C Parmentier M Serhan CN Murphy PM International Union of Basic and Clinical Pharmacology. LXXIII. Nomenclature for the formyl peptide receptor (FPR) family Pharmacol Rev 2009 61 119 161 10.1124/pr.109.001578 19498085 PMC2745437

[B31] John CD Sahni V Mehet D Morris JF Christian HC Perretti M Flower RJ Solito E Buckingham JC Formyl peptide receptors and the regulation of ACTH secretion: targets for annexin A1, lipoxins, and bacterial peptides FASEB J 2007 21 1037 1046 10.1096/fj.06-7299com 17218541 PMC1892899

[B32] Liberles SD Horowitz LF Kuang D Contos JJ Wilson KL Siltberg-Liberles J Liberles DA Buck LB Formyl peptide receptors are candidate chemosensory receptors in the vomeronasal organ Proc Natl Acad Sci USA 2009 106 9842 9847 10.1073/pnas.0904464106 19497865 PMC2690606

[B33] Svensson CI Zattoni M Serhan CN Lipoxins and aspirin-triggered lipoxin inhibit inflammatory pain processing J Exp Med 2007 204 245 252 10.1084/jem.20061826 17242163 PMC2118737

[B34] Tambuyzer BR Ponsaerts P Nouwen EJ Microglia: gatekeepers of central nervous system immunology J Leukoc Biol 2009 85 352 370 19028958 10.1189/jlb.0608385

[B35] Pacher P Beckman JS Liaudet L Nitric oxide and peroxynitrite in health and disease Physiol Rev 2007 87 315 424 10.1152/physrev.00029.2006 17237348 PMC2248324

[B36] Ock J Han HS Hong SH Lee SY Han YM Kwon BM Suk K Obovatol attenuates microglia-mediated neuroinflammation by modulating redox regulation Br J Pharmacol 2010 159 1646 1662 10.1111/j.1476-5381.2010.00659.x 20397299 PMC2925488

[B37] Kaushik DK Gupta M Das S Basu A Kruppel-like factor 4, a novel transcription factor regulates microglial activation and subsequent neuroinflammation J Neuroinflammation 2010 7 68 10.1186/1742-2094-7-68 20946687 PMC2965135

[B38] Jozsef L Zouki C Petasis NA Serhan CN Filep JG Lipoxin A4 and aspirin-triggered 15-epi-lipoxin A4 inhibit peroxynitrite formation, NF-kappa B and AP-1 activation, and IL-8 gene expression in human leukocytes Proc Natl Acad Sci USA 2002 99 13266 13271 10.1073/pnas.202296999 12235371 PMC130622

[B39] Jin SW Zhang L Lian QQ Liu D Wu P Yao SL Ye DY Posttreatment with aspirin-triggered lipoxin A4 analog attenuates lipopolysaccharide-induced acute lung injury in mice: the role of heme oxygenase-1 Anesth Analg 2007 104 369 377 10.1213/01.ane.0000252414.00363.c4 17242094

[B40] Zhao X Bausano B Pike BR Newcomb-Fernandez JK Wang KK Shohami E Ringger NC DeFord SM Anderson DK Hayes RL TNF-alpha stimulates caspase-3 activation and apoptotic cell death in primary septo-hippocampal cultures J Neurosci Res 2001 64 121 131 10.1002/jnr.1059 11288141

[B41] Zou JY Crews FT TNF alpha potentiates glutamate neurotoxicity by inhibiting glutamate uptake in organotypic brain slice cultures: neuroprotection by NF kappa B inhibition Brain Res 2005 1034 11 24 10.1016/j.brainres.2004.11.014 15713255

[B42] Wu SH Liao PY Dong L Chen ZQ Signal pathway involved in inhibition by lipoxin A(4) of production of interleukins induced in endothelial cells by lipopolysaccharide Inflamm Res 2008 57 430 437 10.1007/s00011-008-7147-1 18777114

[B43] Kure I Nishiumi S Nishitani Y Tanoue T Ishida T Mizuno M Fujita T Kutsumi H Arita M Azuma T Yoshida M Lipoxin A(4) reduces lipopolysaccharide-induced inflammation in macrophages and intestinal epithelial cells through inhibition of nuclear factor-kappaB activation J Pharmacol Exp Ther 2010 332 541 548 10.1124/jpet.109.159046 19846590

[B44] Medeiros R Rodrigues GB Figueiredo CP Rodrigues EB Grumman A Jr Menezes-de-Lima O Jr Passos GF Calixto JB Molecular mechanisms of topical anti-inflammatory effects of lipoxin A(4) in endotoxin-induced uveitis Mol Pharmacol 2008 74 154 161 10.1124/mol.108.046870 18413658

[B45] Mattson MP NF-kappaB in the survival and plasticity of neurons Neurochem Res 2005 30 883 893 10.1007/s11064-005-6961-x 16187223

[B46] O'Neill LA Kaltschmidt C NF-kappa B: a crucial transcription factor for glial and neuronal cell function Trends Neurosci 1997 20 252 258 10.1016/S0166-2236(96)01035-1 9185306

[B47] Gewirtz AT Collier-Hyams LS Young AN Kucharzik T Guilford WJ Parkinson JF Williams IR Neish AS Madara JL Lipoxin a4 analogs attenuate induction of intestinal epithelial proinflammatory gene expression and reduce the severity of dextran sodium sulfate-induced colitis J Immunol 2002 168 5260 5267 11994483 10.4049/jimmunol.168.10.5260

[B48] Lu YC Yeh WC Ohashi PS LPS/TLR4 signal transduction pathway Cytokine 2008 42 145 151 10.1016/j.cyto.2008.01.006 18304834

[B49] Wu SH Wu XH Lu C Dong L Zhou GP Chen ZQ Lipoxin A4 inhibits connective tissue growth factor-induced production of chemokines in rat mesangial cells Kidney Int 2006 69 248 256 10.1038/sj.ki.5000025 16408113

[B50] Ohira T Bannenberg G Arita M Takahashi M Ge Q Van Dyke TE Stahl GL Serhan CN Badwey JA A stable aspirin-triggered lipoxin A4 analog blocks phosphorylation of leukocyte-specific protein 1 in human neutrophils J Immunol 2004 173 2091 2098 15265945 10.4049/jimmunol.173.3.2091

[B51] Serhan CN Lipoxins and aspirin-triggered 15-epi-lipoxins are the first lipid mediators of endogenous anti-inflammation and resolution Prostaglandins Leukot Essent Fatty Acids 2005 73 141 162 10.1016/j.plefa.2005.05.002 16005201

[B52] Ereso AQ Cureton EL Cripps MW Sadjadi J Dua MM Curran B Victorino GP Lipoxin a(4) attenuates microvascular fluid leak during inflammation J Surg Res 2009 156 183 188 10.1016/j.jss.2009.01.009 19524267 PMC2946836

[B53] Prieto P Cuenca J Traves PG Fernandez-Velasco M Martin-Sanz P Bosca L Lipoxin A4 impairment of apoptotic signaling in macrophages: implication of the PI3K/Akt and the ERK/Nrf-2 defense pathways Cell Death Differ 2010 17 1179 1188 10.1038/cdd.2009.220 20094061

[B54] Simoes RL Niconi-de-Almeida Y da-Fe AR Barja-Fidalgo C Fierro IM A synthetic analog of 15-epi-lipoxin A4 inhibits human monocyte apoptosis: involvement of ERK-2 and PI3-kinase Prostaglandins Other Lipid Mediat 2010 91 10 17 10.1016/j.prostaglandins.2009.12.001 20004734

